# The impact of DRG on resource consumption of inpatient with ischemic stroke

**DOI:** 10.3389/fpubh.2023.1213931

**Published:** 2023-11-07

**Authors:** Anle Wei, Jianing Ren, Wen Feng

**Affiliations:** Department of Health Policy and Management, School of Public Health, Peking University, Beijing, China

**Keywords:** DRG payment reform, interrupted time series, ischemic stroke, policy evaluation, China

## Abstract

**Background:**

Diagnosis-related group (DRG) payments were gradually introduced and used in 12 public hospitals in L city. Given the high incidence and burden of ischemic stroke, the study aimed to assess the impact of DRG payment reform on inpatient medical resource utilization.

**Methods:**

Data were obtained from the DRG local database of the new Chinese cooperative medical program in L city. The study used interrupted time series analysis to examine changes in length of stay and medical costs before and after the reform, and also assessed changes in different subgroups.

**Results:**

There were 763 and 4,731 ischemic stroke patients in tertiary hospitals and 1953 and 10,439 patients in secondary hospitals before and after the DRG payment reform, respectively. After the reform, LOS was reduced 0.047 and 0.47 days in tertiary and secondary hospitals, respectively. Medical expenses decreased by 30.189 yuan in tertiary hospitals, but those increased by 44.918 yuan in secondary hospitals monthly. For gender, the average LOS reduced by 0.462 and 0.471 days for male and female in secondary hospitals. The change in medical expenses for male patients in tertiary hospitals and female in secondary hospitals were more significant, with a decrease of 65.396 yuan and increase of 56.257 yuan. The most pronounced change in resource consumption was seen for patients aged 85 years and older, with an increase in average LOS and medical expenses by 0.394 days and 382.422 yuan in tertiary hospitals. They showed a reduction in the average LOS by 1.480 days, and increase in the average medical expenses by 133.485 yuan in secondary hospitals monthly. Regarding disease severity, the most significant changes were seen in MCC patients. The average LOS decreased by 0.197 and 0.928 days and the average medical expenses decreased by 131.526 and 21.631 yuan in tertiary and secondary hospitals, respectively.

**Conclusion:**

The implementation of DRG payment system has led to a reduction in the LOS in various levels of hospitals, which would save in bed resources. However, DRG payment reform can help to control medical expenses for ultra-high cases, but it may not be useful to control the overall increase in medical expenses.

## 1. Introduction

Diagnosis-related groups (DRGs) have become a vital tool for regulating medical practice in many countries. The United States was the first country to implement DRG payment reform in the 1970s, and subsequent studies found that hospitals subject to the all-payer DRG system had lower increases in cost per admission and cost per day than those institutions that were reimbursed retrospectively (1). As a result, many countries and regions, including Australia, Portugal, Ireland, France, Germany and so on, adopted the DRG approach for inpatient care to control spending and improve transparency, efficiency, and quality of healthcare (2).

Chinese scholars have been interested in DRGs since the late 1980s and have developed several local versions of DRGs in China (3). However, these DRGs were not implemented nationwide due to the immature conditions in terms of information standardization, disease coding technology, and cost management at that time (4). With the aging of China, it is difficult to maintain the short-term and long-term balance of income and expenditure of medical insurance, and the introduction of DRG management tools was considered. During a pilot process, research showed that DRG payment resulted in a reduction of approximately 6.2% in medical expenditure per admission and approximately 10.5% in out-of-pocket expenses (5). Scholars have reviewed the resource consumption after the introduction of DRG and concluded that China’s localized DRG-based payments may slightly improve efficiency and may save medical expenses by reducing the average length of stay (LOS) (6). However, the equity and quality of healthcare may be compromised, such as the tendency of hospitals to avoid critically ill or older adult patients (7). More and more cities in China launched DRG payment for social health insurance scheme. There is essential to provide evidences of usability, applicability and its effect on equity of localized DRG version of for policy-makers and hospitals managers.

Stroke is the second leading cause of death worldwide and a significant cause of disability, with an increasing incidence in developing countries (8). In China, ischemic stroke or transient ischemic attack (TIA) is the most common type of cerebrovascular event and accounts for approximately 70% of all strokes (9), leading to significant disability among patients and a heavy burden on families and the country as a whole (10). Research by Wang et al. has indicated that the aging population plays a vital role in long-term trends in ischemic stroke morbidity and mortality, with the older adult population remaining the primary source of the stroke burden (11).

While several studies have explored the medical expenses of ischemic stroke in developed countries (12, 13), there is a lack of evidence regarding DRG and ischemic stroke in developing countries, and few studies have investigated the resource consumption of ischemic stroke patients among diverse population groups (14, 15). Therefore, this study aims to evaluate the impact of DRG payment reform policies on resource consumption among inpatients with ischemic stroke, and compare its influence on different population subgroups across hospital grades.

## 2. Methods

### 2.1. Data sources

L City, located in Southwest China, is an important railway hub and logistics distribution center. It was selected as one of the pilot cities for the China’s DRG for New Cooperative Medical Scheme (CR-DRG) reform, which was initiated in October 2017 by four public hospitals. Subsequently, the second and third batches of hospitals were included in the reform in January and April 2018, respectively, covering a total of 12 acute hospitals. The main aim of the reform was to effectively control the unreasonable rise of medical expenses and reduce the financial burden on patients (16). Following the implementation of DRG, all 12 public hospitals in L City began using it for inpatient payment, making it the first city in China to have all its public hospitals covered by DRG. This research focuses on analyzing the impact of medical resource consumption after the implementation of DRG in L City, particularly on patients with ischemic stroke, given its high incidence and burden. It is worth noting that there are three levels of public hospitals in China, namely tertiary, secondary, and primary hospitals, but DRG is only implemented in tertiary and secondary hospitals as there is minimal inpatient service in primary providers.

The study collected data on hospitalized patients with ischemic stroke (DRG code BR29) from January 1, 2017, to December 31, 2020, from the CR-DRG database of the local health insurance administration in L City. The data were obtained from 4 tertiary hospitals and 8 secondary hospitals and included information such as demographic characteristics, diagnosis, essential treatment process, DRGs, and medical expenses (in RMB yuan). In the study, average length of hospital stay and average hospitalization cost were used as dependent indicators. In conjunction with previous studies, the independent variables included personal characteristics of patients such as gender and age and characteristics of the treatment model such as admission route, mode of discharge, and other diagnosed diseases severity. Combined with the definition of aging and the WHO age classification criteria for age grouping, the age of the patients was divided into three segments: old (≥65 years old), middle (45–64 years old), young (≤44 years old), and the older adult over 65 years old were divided into the young older adult, the old older adult, and the older adult with longevity using the cut-off point of 75 years old and 85 years old. Other diagnoses of disease severity were categorized as major comorbidities or complications (MCC), general comorbidities or complications (CC), and no comorbidities or complications, admission routes included outpatient admissions and others, and discharge modes included medical discharge and others. The flowchart illustrating the study design is presented in Supplementary Figure 1.

### 2.2. Data analysis

The study utilized Interrupted Time Series (ITS) analysis, which is a quasi-experimental research design commonly used in evaluating public health interventions. The ITS analysis covered a time series from January 2017 to December 2020, with 10 months before the reform (January to October 2017) and 20 months after the reform (April 2018 to December 2020). The intermediate period was from October 2017 to April 2018 when the 12 hospitals gradually transferred from Fee-for-service (FFS) to DRG payment programs. So, the 48 months of observations included three processes, before the reform, the transition period and after the reform. Since there are differences between tertiary and secondary public hospitals in China in terms of resource conditions, treatment, and regulatory cost expenditures, separate ITS analyses were conducted for each.

The ITS analysis was used to estimate the instantaneous (change in outcome level) and long-term (change in outcome trend) impact of the CR-DRG policy.

The study utilized a piecewise regression model with the following formula:


$$Y_{t}=\beta_{0}+\beta_{1}\times X_{\text{time}}+\beta_{2}\times X_{DRG}+\beta_{3}\times X_{\text{posttime}}+e_{t}$$

where Y_t_ is the research outcome variable at time t; time refers to a continuous time variable; in this study, the time unit is months; *X*_DRG_ refers to the dummy variable before intervention (coded as 0) or after intervention (coded as 1); β_0_ is the estimated value of the baseline level *t* = 0; β_1_ is the change in outcome associated with an increase in the time unit (representing the trend before the intervention); β_2_ is the estimated value of the instantaneous level change; and β_3_ is represents the trend change; β_1_ + β_3_ represents the trend after the intervention; e_t_ refers to the error term. STATA16.0 software was used for data analysis.

## 3. Results

### 3.1. Basic information of patients

A descriptive analysis was conducted to examine the characteristics of ischemic stroke patients before (January 2017–October 2017) and after (April 2018–December 2020) the DRG payment reform in L City. There were 763 and 4,731 ischemic stroke patients admitted to tertiary hospitals before and after the reform, respectively. In secondary hospitals, there were 1953 and 10,439 ischemic stroke patients before and after the reform, respectively. Among different grades of hospitals, patients aged 65 years or older accounted for approximately 50% or more, and the average medical expenses increased gradually with age. The proportion of female patients was slightly higher than that of male patients. Outpatient admissions accounted for about 70–80% of the total number of patients, and patients discharged from the hospital on medical orders accounted for more than 85% of the total. Patients with disease severity of CC had the highest proportion, and the average medical expenses increased as the disease severity increased (Supplementary Tables 1, 2).

### 3.2. Interrupted time series analysis of the average length of stay of inpatients with ischemic stroke

The ITS results indicated that the baseline LOS of tertiary hospitals and secondary hospitals were 12.849 days and 7.744 days (*p* < 0.001). Before the introduction of DRG, the LOS in secondary hospitals decreased significantly, with an average decrease of approximately 0.366 days per month (*p* < 0.05). After the introduction of the DRG policy, the β3 values of tertiary and secondary hospitals were −0.039 (*p* < 0.05) and −0.104 (*p* < 0.001), respectively, indicating that the policy had a consistent and significant impact on reducing the LOS in both levels of hospitals. The slopes for tertiary and secondary hospitals were −0.047 and −0.47 after the implementation of DRG, respectively, indicating that the decrease in LOS was more significant in secondary hospitals. The results suggest that the DRG payment policy had a greater impact on reducing the average LOS for ischemic stroke patients in secondary hospitals compared to tertiary hospitals (Supplementary Table 3; Figure 2).

### 3.3. Interrupted time series analysis of the average medical expenses of inpatients with ischemic stroke

During the study period, the baseline medical expenses of tertiary hospitals and secondary hospitals were 6466.419 yuan and 4328.498 yuan, respectively, and both were significantly different (*p* < 0.001). After the implementation of the policy, the β3 values of tertiary and secondary hospitals were 7.324 and 26.286, respectively, but only secondary hospitals showed statistical significance (*p* < 0.001). Overall, the slopes for tertiary and secondary hospitals were −30.189 and 44.918 after the implementation of the DRG intervention, respectively. This represents a decrease of 30.189 yuan per month in medical expenses for tertiary hospitals and an increase of 44.918 yuan for secondary hospitals (Supplementary Table 3; Figure 3). The implementation of the DRG policy had a greater impact on the medical expenses of secondary hospitals, which showed a significant trend of substantial growth.

### 3.4. Impact of individual characteristics on length of stay before and after diagnosis-related group reform

#### 3.4.1. Impact of gender subgroup

At baseline, the average LOS for male and female ischemic stroke patients was 8.090 and 7.463 days for tertiary hospitals, and 12.697 and 12.934 days for secondary hospitals, respectively (*p* < 0.001). Before the implementation of the DRG policy, only secondary hospitals had a statistically significant decrease in the LOS per month for male and female patients, with β1 values of −0.349 and −0.373, respectively (*p* < 0.05). In terms of trend change, the β3 for female ischemic stroke patients in tertiary hospitals was −0.038 (*p* < 0.10), while it was −0.113 for male patients and −0.098 for female patients in secondary hospitals, both of which were statistically significant (*p* < 0.001). After the formal implementation of the DRG policy, the average LOS was reduced by 0.089, 0.003, 0.462, and 0.471 days for male and female ischemic stroke patients in tertiary hospitals and male and female ischemic stroke patients in secondary hospitals, respectively (Supplementary Table 4; Figure 4).

#### 3.4.2. Impact of age subgroup

The average LOS for ischemic stroke patients in different age groups was analyzed in this study. The results showed that the baseline average LOS for ischemic stroke patients aged 0–44 years, 45–64 years, 65–74 years, 75–84 years, and 85 years and older was 6.199, 7.481, 8.497, 7.526, and 7.257 days in tertiary hospitals, respectively, and 9.859, 13.105, 13.505, and 11.479 days and 18.032 days in secondary hospitals, and the above were statistically significant (*p* < 0.001). Before the reform of the DRG payment system, patients aged 44–64 years had a statistically significant (*p* < 0.001) reduction in LOS of 0.454 days per month in secondary hospitals, while other age groups showed no significant difference (*p* > 0.05). In terms of trend changes, patients aged 65–74 and 75–84 years had a slight reduction in the average LOS in tertiary hospitals of 0.049 and 0.050 days monthly (*p* < 0.05). The average LOS was reduced by 0.090, 0.106, 0.106, and 0.100 days for patients in the 0–44, 44–64, 65–74, and 75–84 age groups monthly in the secondary hospital, respectively (*p* < 0.05). Overall, the implementation of the DRG intervention resulted in the greatest reduction in the average LOS in tertiary hospitals for patients aged 65–74 years, with a reduction of 0.159 days per month. In contrast, the most significant change in the average LOS was observed in patients aged 85 years or older, and the change was in the opposite direction in tertiary and secondary hospitals, with an increase of 0.394 days per month in tertiary hospitals and a decrease of 1.480 days per month in secondary hospitals (Supplementary Table 4; Figure 5).

#### 3.4.3. Impact of disease severity subgroup

Namely, disease severity was classified into three grades: MCC, CC, and none. At baseline, the average LOS for stroke patients with MCC, CC, and no comorbidities or complications in tertiary hospitals were 8.806, 8.733, and 5.783 days, respectively, with statistically significant differences (*p* < 0.001). For secondary hospitals, the corresponding figures were 15.735, 13.495, and 11.174 days, also with statistically significant differences (*p* < 0.001). After the DRG policy was implemented, stroke patients with MCC, CC, and no comorbidities or complications in tertiary hospitals experienced a reduction of 0.197, 0.131, and 0.124 days per month, respectively. In secondary hospitals, the corresponding reductions were 0.928, 0.482, and 0.343 days per month, respectively (Supplementary Table 4; Figure 6).

### 3.5. Impact of individual characteristics on medical expenses before and after diagnosis-related group reform

#### 3.5.1. Impact of gender subgroup

The baseline average medical expenses for ischemic stroke patients in tertiary hospitals were 7,001.463 yuan and 6,029.202 yuan for male and female patients, respectively. In secondary hospitals, the corresponding figures were 4,654.529 yuan and 4,128.745 yuan for male and female patients, respectively, and all of these differences were statistically significant (*p* < 0.001). Before the implementation of the DRG policy, only the β1 of the male patient group in the tertiary hospital was statistically significant (*p* < 0.05), with a value of −79.131. In terms of trend changes, in secondary hospitals, the β3 was statistically significant (*p* < 0.001), with a value of 36.044 for male patients and 20.025 for female patients. After the introduction of DRG, the average medical expenses for male stroke patients in tertiary hospitals decreased by 65.396 yuan monthly, while for female patients in tertiary hospitals, it increased by 25.289 yuan. The corresponding figures in secondary hospitals were a decrease of 15.727 yuan and an increase of 56.267 yuan for male and female stroke patients, respectively (Supplementary Table 5; Figure 7).

#### 3.5.2. Impact of age subgroup

In terms of age comparison, the study found that the baseline medical expenses for ischemic stroke patients varied significantly among different age groups. Specifically, in tertiary hospitals, the average medical expenses were 4442.705, 6321.810, 7140.566, 6156.669, and 6635.419 yuan for patients aged 0–44 years, 45–64 years, 65–74 years, 75–84 years, and 85 years and above, respectively. The corresponding figures for secondary hospitals were 3520.251, 4389.832, 4547.869, 4149.152, and 3843.815 yuan, respectively. All of these differences were statistically significant (*p* < 0.001). Before the implementation of DRG, the average medical expenses of patients aged 44–64 years and 65–74 years decreased by 66.704 and 145.346 yuan in tertiary hospitals, respectively. However, the average medical expenses of patients aged 75–84 years increased by 102.458 yuan in secondary hospitals, and this increase was statistically significant (*p* < 0.05). In terms of trend changes, only the medical expenses of patients aged 0–44 years in tertiary hospitals showed a statistically significant increase of 64.146 yuan per month on average (*p* < 0.05). However, the medical expenses of all age groups in secondary hospitals showed an increasing trend, and all of them were statistically significant (*p* < 0.05). Overall, after the implementation of DRG intervention, the average medical expenses in tertiary hospitals decreased the most for patients aged 65–74 years, by 126.411 yuan per month. On the other hand, patients aged 85 years and above showed the most significant changes, with an increase of 382.422 yuan per month in average medical expenses in tertiary hospitals and an increase of 133.485 yuan per month in secondary hospitals (Supplementary Table 5; Figure 8).

#### 3.5.3. Impact of disease severity subgroup

The average medical expenses for ischemic stroke patients in tertiary hospitals were 7966.282, 7216.951, and 4752.744 yuan for those with the highest to lowest disease severity, respectively. In secondary hospitals, the expenses were 5171.367, 4562.963, and 3774.441 yuan, respectively. All results showed significant statistical differences (*p* < 0.001). In terms of trend changes, the medical expenses of patients with MCC, CC, no complications, and complications in secondary hospitals increased by 39.840, 21.883, and 31.516 yuan per month, respectively, and the differences were statistically significant (*p* < 0.01). Overall, after the implementation of DRG intervention, the medical expenses of MCC or CC patients in tertiary hospitals, and MCC patients in secondary hospitals all showed a downward trend, indicating that the medical expenses of patients with higher disease severity decreased after DRG reform (Supplementary Table 5; Figure 9).

## 4. Discussion

This study investigated the impact of the DRG payment system on healthcare resource utilization for ischemic stroke inpatients in L city, China. The results indicated that the DRG payment system increased hospital efficiency, as demonstrated by a reduction in the LOS for both tertiary and secondary hospitals. Changes of LOS from FFS to DRG are consistent with previous studies conducted in Europe and Asia (17, 18), which also reported a decrease in LOS following the implementation of DRGs. However, the study also revealed that patient hospital expenses did not decrease after the introduction of the DRG payment system. On the contrary, they continued to be higher, consistent with some studies from other countries (19, 20). Notably, a study conducted in Zhongshan city, China, found a trend toward lower medical expenses following the implementation of DRGs (21). The impact of DRG payment systems on controlling medical expenses is a complex issue that may be influenced by factors such as city, hospital, or disease type. Therefore, further research is needed to monitor and evaluate the effects of DRG payment systems on healthcare resource utilization in China (13).

Medical resource consumption at different levels hospitals tend to change in DRG payment reform differently. Secondary hospitals showing more significant compression of LOS and control of medical expenses than tertiary hospitals, as shown in a previous study conducted in Poland (22). The differential change of LOS and medical expenses between tertiary and secondary hospitals, with secondary hospitals demonstrating statistically significant differences in most patient subgroups, while tertiary hospitals did not. This suggests that the implementation of DRG policy had varying impacts on healthcare resource consumption in different hospital settings. The higher level of hospitals, more serious inpatients, higher medical consumption before DRG reform is essential necessary but controllable (23). In addition, management capital of tertiary hospitals is much more effective than secondary hospitals before the DRG reform (24). So that those tertiary hospitals have less opportunity and possibility to narrow down the LOS than secondary hospitals.

The study suggests that the DRG payment system has varying effects on patients with different severity levels across different hospital levels. Specifically, after DRG implementation, the medical expenses of patients with MCC or CC in tertiary hospitals, and MCC patients in secondary hospitals, exhibited a decreasing trend. This indicates that DRG has prompted hospitals to reduce unnecessary medical interventions relative to FFS system, but there is a need to be wary of underservicing (25). However, medical expenses for CC patients in secondary hospitals increased significantly, indicating potential upcoding behavior that requires monitoring. Previous research has shown that such behavior may be driven by pressure from administrators and professionals (26). Therefore, when evaluating the effects of the DRG payment system, it is important to analyze overall situations and different specific indicators.

After the implementation of the DRG payment system, older adult patients, especially those who are long-lived, continue to consume higher levels of health resources. The study found that the average LOS and medical expenses of patients under 75 years of age in tertiary hospitals showed a decreasing trend after the implementation of the policy, while increasing for all patients over 75 years of age. The largest decrease in average LOS and the highest increase in average medical expenses were observed among patients aged 85 years or older in secondary hospitals. One possible explanation is that older patients may have more complex health needs and require more frequent and intensive care (27), which may lead to increased healthcare expenses. Also, patients under 75 years of age presented the effect of DRG reforms more toward the desired goal, suggesting that DRG implementation is more conducive to a rational distribution of health care resources among patients of different ages (28). It also further suggests that the implementation of a DRG system requires differentiated measures for specific patient subgroups.

There were other aspects of this study that differed from the results of previous studies. Notably, male patients in tertiary hospitals experienced a decrease in medical expenses following DRG implementation, while female patients in tertiary hospitals and both male and female patients in secondary hospitals continued to experience increasing medical expenses. These results differ from a previous study conducted in China that did not identify a significant impact of gender on in-hospital expenses for DRG-based stroke patients (29).

## 5. Conclusion

This study sheds light on the impact of the DRG payment system on healthcare resource utilization for inpatients with ischemic stroke in China. The findings indicate that the DRG payment system led to improved efficiency in bed utilization, as evidenced by the decrease in the average LOS for patients. There was no reduction in medical expenses overall, while medical expenses decreased for the highest-spending patients in each subgroup. This suggests that the DRG payment reform was beneficial in controlling medical expenses for ultra-high cases. However, expenses continued to increase for some patients, especially those with complex medical needs and older patients. This suggests that the implementation of the DRG payment system needs to be tailored to specific patient subgroups. Due to the lag effect of the policy, further research is needed to evaluate the long-term impact of this payment reform on healthcare expenses for patients with ischemic stroke. To improve the credibility of the findings, future studies should consider increasing the sample size and expanding the evaluation metrics. This study also has some limitations. First, the financial burden on patients and their families was underestimated because the cost of prognostic care and rehabilitation of the disease and related indirect costs were not estimated and price increases and inflation over a 4-year period were not taken into account. In addition, there were some personal factors close to the clinic, such as whether the patients had diabetes, hypertension, hyperlipidemia and other diseases, a history of smoking or a history of alcohol consumption, which were not included considering the available data.

## Data availability statement

The data analyzed in this study is subject to the following licenses/restrictions: confidential data. Requests to access these datasets should be directed to WF, fengw@hsc.pku.edu.cn.

## Author contributions

AW designed the study and wrote a manuscript. JR has revised the manuscript. WF has revised, edited, and corrected the article. All authors contributed to the article and approved the submitted version.

## References

[1] Rosko MD Broyles RW. Short-term responses of hospitals to the DRG prospective pricing mechanism in New Jersey. Med Care. (1987) 25:88–99. doi: 10.1097/00005650-198702000-00002, PMID: 3102862

[2] Zou K Li HY Zhou D Liao ZJ. The effects of diagnosis-related groups payment on hospital healthcare in China: a systematic review. BMC Health Serv Res. (2020) 20:112. doi: 10.1186/s12913-020-4957-5, PMID: 32050962 PMC7017558

[3] Liang X Guo H Jin C Peng X Zhang X. The effect of new cooperative medical scheme on health outcomes and alleviating catastrophic health expenditure in China: a systematic review. PloS One. (2012) 7:e40850. doi: 10.1371/journal.pone.0040850, PMID: 22916098 PMC3423411

[4] Wang Z Liu R Li P Jiang C Hao M. How to make diagnosis related groups payment more feasible in developing countries-a case study in Shanghai, China. Iran J Public Health. (2014) 43:572–8. 26060758 PMC4449405

[5] Jian W Lu M Chan KY Poon AN Han W Hu M. The impact of a pilot reform on the diagnosis-related-groups payment system in China: a difference-in-difference study. Lancet. (2015) 386:S26. doi: 10.1016/S0140-6736(15)00604-2

[6] Meng Z Hui W Cai Y Liu J Wu H. The effects of DRGs-based payment compared with cost-based payment on inpatient healthcare utilization: a systematic review and meta-analysis. Health Policy. (2020) 124:359–67. doi: 10.1016/j.healthpol.2020.01.007, PMID: 32001043

[7] Rosenthal GE Landefeld CS. Do older Medicare patients cost hospitals more?: evidence from an Academic Medical Center. Arch Intern Med. (1993) 153:89–96. doi: 10.1001/archinte.1993.00410010111010 8422203

[8] Campbell BCV De Silva DA Macleod MR Coutts SB Schwamm LH Davis SM. Ischaemic stroke. Nat Rev Dis Primers. (2019) 5:70. doi: 10.1038/s41572-019-0118-8 31601801

[9] Wang Y Jing J Meng X Pan Y Wang Y Zhao X. The third China National Stroke Registry (CNSR-III) for patients with acute ischaemic stroke or transient ischaemic attack: design, rationale and baseline patient characteristics. Stroke Vasc Neurol. (2019) 4:158–64. doi: 10.1136/svn-2019-000242, PMID: 31709123 PMC6812638

[10] Lekander I Willers C von Euler M Lilja M Sunnerhagen KS Pessah-Rasmussen H. Relationship between functional disability and costs one and two years post stroke. PloS One. (2017) 12:e0174861. doi: 10.1371/journal.pone.0174861, PMID: 28384164 PMC5383241

[11] Wang Y Zhou L Guo J Wang Y Yang Y Peng Q. Secular trends of stroke incidence and mortality in China, 1990 to 2016: the global burden of disease study 2016. J Stroke Cerebrovasc Dis. (2020) 29:104959. doi: 10.1016/j.jstrokecerebrovasdis.2020.104959, PMID: 32689583

[12] Demaerschalk BM Durocher DL. How diagnosis-related group 559 will change the US Medicare cost reimbursement ratio for stroke centers. Stroke. (2007) 38:1309–12. doi: 10.1161/01.STR.0000260185.74694.a7, PMID: 17332446

[13] Eyding J Misselwitz B Weber R Neumann-Haefelin T Bartig D Krogias C. Vergleichbarkeit unterschiedlicher Datenquellen zur Schlaganfallversorgung in Deutschland. Nervenarzt. (2020) 91:877–90. doi: 10.1007/s00115-020-00989-8, PMID: 32930815

[14] Mathauer I Wittenbecher F. Hospital payment systems based on diagnosis-related groups: experiences in low-and middle-income countries. Bull World Health Organ. (2013) 91:746–756A. doi: 10.2471/BLT.12.115931, PMID: 24115798 PMC3791650

[15] Brügger U Eichler K. Impact of introducing a DRG reimbursement system in an acute impatient hostpital setting: a literature review. Dublin, Ireland: HTAi 7th Annual Meeting (2010).

[16] Zhai T Goss J Li J. Main drivers of health expenditure growth in China: a decomposition analysis. BMC Health Serv Res. (2017) 17:185. doi: 10.1186/s12913-017-2119-1, PMID: 28274228 PMC5343399

[17] Busse R Geissler A Aaviksoo A Cots F Häkkinen U Kobel C. Diagnosis related groups in Europe: moving towards transparency, efficiency, and quality in hospitals? BMJ. (2013) 346:f 3197. doi: 10.1136/bmj.f3197, PMID: 23747967

[18] Annear PL Kwon S Lorenzoni L Duckett S Huntington D Langenbrunner JC. Pathways to DRG-based hospital payment systems in Japan, Korea, and Thailand. Health Policy. (2018) 122:707–13. doi: 10.1016/j.healthpol.2018.04.013, PMID: 29754969

[19] Magnussen J Hagen TP Kaarboe OM. Centralized or decentralized? A case study of Norwegian hospital reform. Soc Sci Med. (2007) 64:2129–37. doi: 10.1016/j.socscimed.2007.02.018 17368681

[20] Mikkola H Keskimäki I Häkkinen U. DRG-related prices applied in a public health care system—can Finland learn from Norway and Sweden? Health Policy. (2002) 59:37–51. doi: 10.1016/S0168-8510(01)00169-5, PMID: 11786173

[21] Yuan S Liu W Wei F Zhang H Wang S Zhu W. Impacts of hospital payment based on diagnosis related groups (DRGs) with global budget on resource use and quality of care: a case study in China. Iran J Public Health. (2019) 48:238. doi: 10.18502/ijph.v48i2 31205877 PMC6556178

[22] Buczak-Stec E Goryński P Nitsch-Osuch A Kanecki K Tyszko P. The impact of introducing a new hospital financing system (DRGs) in Poland on hospitalisations for atherosclerosis: an interrupted time series analysis (2004–2012). Health Policy. (2017) 121:1186–93. doi: 10.1016/j.healthpol.2017.09.009 28967491

[23] Mendez CM Harrington DW Christenson P Spellberg B. Impact of hospital variables on case mix index as a marker of disease severity. Popul Health Manag. (2014) 17:28–34. doi: 10.1089/pop.2013.0002, PMID: 23965045 PMC3931432

[24] Dong S Millar R Shi C Dong M Xiao Y Shen J. Rating hospital performance in China: review of publicly available measures and development of a ranking system. J Med Internet Res. (2021) 23:e17095. doi: 10.2196/17095, PMID: 34137724 PMC8277410

[25] Jiang G Peng Q. Medical payment series: The rise of the DRG payment model. Milliman White Paper (2019). 1–8 p.

[26] Georgescu I Hartmann FGH. Sources of financial pressure and up coding behavior in French public hospitals. Health Policy. (2013) 110:156–63. doi: 10.1016/j.healthpol.2013.02.003, PMID: 23477807

[27] Chen RL Balami JS Esiri MM Chen LK Buchan AM. Ischemic stroke in the elderly: an overview of evidence. Nat Rev Neurol. (2010) 6:256–65. doi: 10.1038/nrneurol.2010.36, PMID: 20368741

[28] Muñoz E Rosner F Chalfin D Goldstein J Margolis IB Wise L. Financial risk and hospital cost for elderly patients: age-and non—age-stratified medical diagnosis related groups. Arch Intern Med. (1988) 148:909–12. doi: 10.1001/archinte.1988.00380040149021 3128196

[29] Qiao D Zhang Y Rehman A Khosravi MR. Big data-enabled analysis of DRGs-based payment on stroke patients in Jiaozuo, China. Shahzad S, editor. J Healthcare Engineer. 2020. (2020)::6690019. doi: 10.1155/2020/6690019, PMID: 33343852 PMC7728475

