# Supplementary material for: The impact of DRG on resource consumption of inpatient with ischemic stroke
Source: Front Public Health. 2023 Nov 7;11:1213931. doi: 10.3389/fpubh.2023.1213931 (PMC10662082; doi:10.3389/fpubh.2023.1213931)
Supplement: Supplementary file 1 [file Table_1.docx]

Supplementary Material

The impact of DRG on resource consumption of inpatient with ischemic stroke

Anle Wei , Jianing Ren, Wen Feng*

*** Correspondence:** Wen Feng: fengw@hsc.pku.edu.cn

# Supplementary Figures and Tables

## Supplementary Figures

##
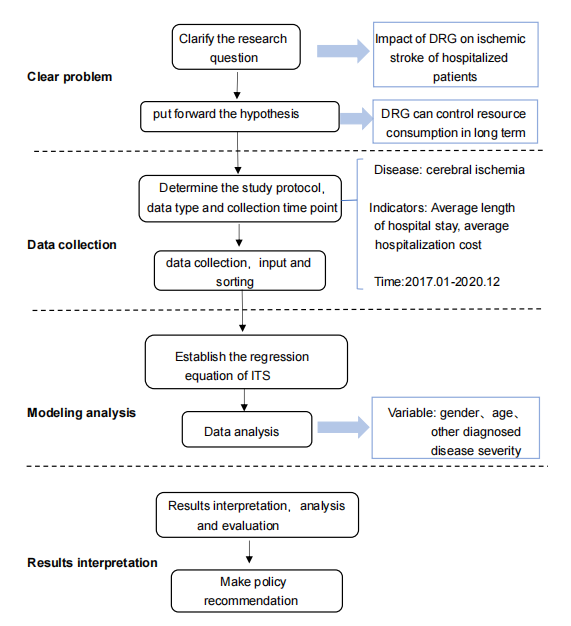


## Supplementary Figure 1. Flowchart of the analysis of resource consumption in ischemic stroke patients.

**Supplementary Figure 2.** Estimated change in LOS in tertiary hospitals and secondary hospitals.

**Supplementary Figure 3.** Estimated change in medical expenses in tertiary hospitals and secondary hospitals.

(a)

(b)

**Supplementary Figure 4.** Estimated changes in the LOS for patients in tertiary hospitals (a) and secondary hospitals (b) by gender. On the left is male, right is female.

(a)

(b)

**Supplementary Figure 5.** Estimated changes in the LOS for patients in tertiary hospitals (a) and secondary hospitals (b) by age. From left to right: under 44 group, 44-64 group, 65-74 group, 75-84 group, and over 85 groups

(a)

(b)

**Supplementary Figure 6.** Estimated changes in the LOS for patients in tertiary hospitals (a) and secondary hospitals (b) by disease severity. On the left is the “MCC” group, in the middle is the “CC” group, and on the right is the “None” group.

(a)


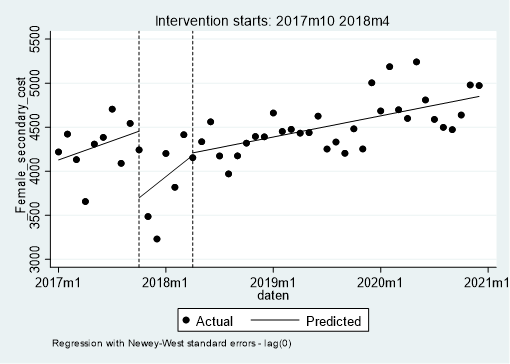


(b)

**Supplementary Figure 7.** Estimated changes in medical expenses for patients in tertiary hospitals (a) and secondary hospitals (b) by gender. On the left is male, right is female.

(a)

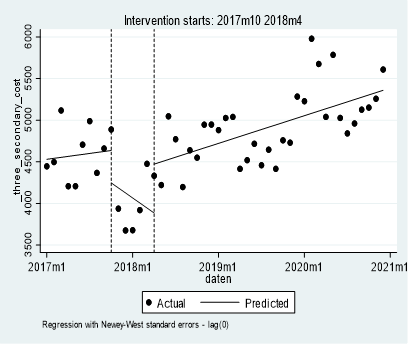

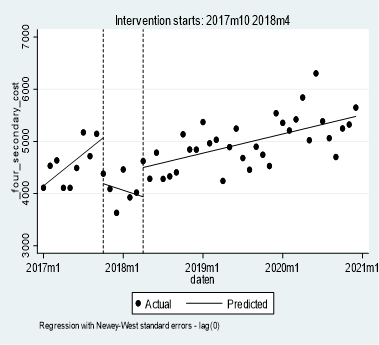

(b)

**Supplementary Figure 8.** Estimated changes in medical expenses for patients in tertiary hospitals (a) and secondary hospitals (b) by age. From left to right: under 44 group, 44-64 group, 65-74 group, 75-84 group and over 85 group.


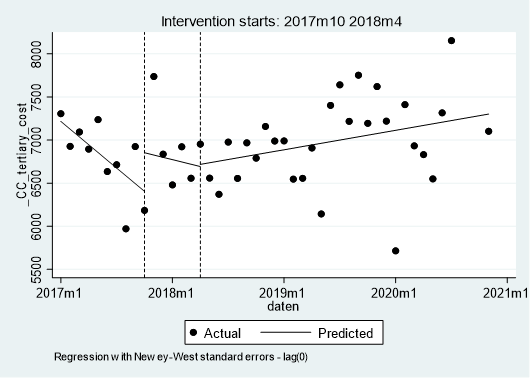


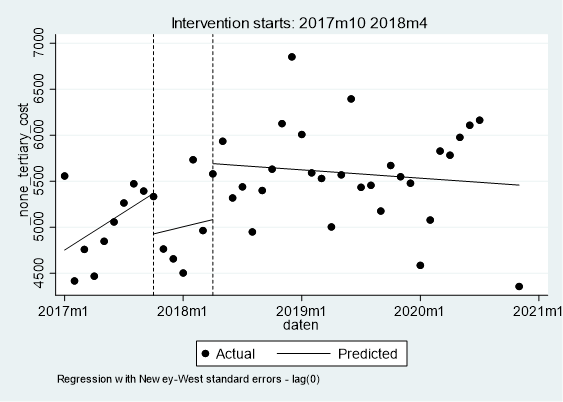


(a)

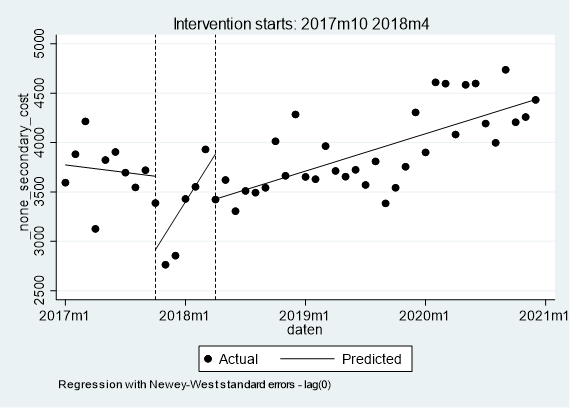


(b)

**Supplementary Figure 9.** Estimated changes in medical expenses for patients in tertiary hospitals (a) and secondary hospitals (b) by disease severity. On the left is the “MCC” group, in the middle is the “CC” group, and on the right is the “None” group.

## Supplementary Tables

**Supplementary Table 1.** The hospitalization volume and medical expenses of ischemic stroke before and after the reform in tertiary hospitals

| Indicators | Before the reform of tertiary hospitals | | |  | After the reform of tertiary hospitals | | |  |
| --- | --- | --- | --- | --- | --- | --- | --- | --- |
|  | No. of patients/case | Average cost per visit (x±s)/RMB | *F*-value |  | No. of patients /case | Average cost per visit (x±s)/RMB | *F*-value |  |
|  |  |  |  |  |  |  |  |  |
| Age group |  |  |  |  |  |  |  |  |
| ≤44 | 58 | 5580.74±3237.03 | 4.65^**^ |  | 330 | 6074.71±4550.84 | 12.68^***^ |  |
| 45- | 304 | 6858.77±4062.01 |  |  | 1942 | 7275.87±4549.42 |  |  |
| 65- | 233 | 7339.43±4714.55 |  |  | 1363 | 7968.27±7166.12 |  |  |
| 75- | 142 | 7974.31±4832.91 |  |  | 981 | 8123.12±4747.59 |  |  |
| ≥85 | 26 | 8935.90±4107.90 |  |  | 115 | 8567.69±5807.38 |  |  |
| Gender |  |  |  |  |  |  |  |  |
| Female | 445 | 6723.55±3992.32 | 11.95^**^ |  | 2666 | 7241.12±5934.77 | 25.71^***^ |  |
| Male | 318 | 7835.04±4869.96 |  |  | 2065 | 8060.24±4909.58 |  |  |
| Admission Route |  |  |  |  |  |  |  |  |
| Others | 69 | 5767.20±4123.05 | 7.93^**^ |  | 600 | 9863.72±11028.21 | 118.35^***^ |  |
| Outpatient | 694 | 7327.93±4416.15 |  |  | 4131 | 7269.66±4058.29 |  |  |
| Discharge Route |  |  |  |  |  |  |  |  |
| Others | 49 | 5870.64±3890.94 | 4.69^*^ |  | 303 | 7494.01±9612.12 | 0.12 |  |
| Medical discharge | 714 | 7277.12±4432.23 |  |  | 4427 | 7606.27±5130.17 |  |  |
| Disease Severity |  |  |  |  |  |  |  |  |
| None | 240 | 5111.83±2442.88 | 52.31^***^ |  | 1473 | 6081.02±3442.29 | 100.61^***^ |  |
| CC | 430 | 7794.83±4391.09 |  |  | 2729 | 8044.79±5056.44 |  |  |
| MCC | 93 | 9730.15±6018.08 |  |  | 529 | 9522.94±9858.02 |  |  |

*: P<0.05; **: P<0.01; ***: P<0.001.

**Supplementary Table 2.** The hospitalization volume and medical expenses of ischemic stroke before and after the reform in secondary hospitals

| Indicators | | Before the reform of secondary hospitals | | | | | |  | | After the reform of secondary hospitals | | | | |  |
| --- | --- | --- | --- | --- | --- | --- | --- | --- | --- | --- | --- | --- | --- | --- | --- |
|  |  | No. of patients/case | | | Average cost per visit (x±s)/RMB | | *F*-value |  | | No. of patients/case | | Average cost per visit (x±s)/RMB | | *F*-value |  |
|  |  |  |  |  |  |  |  |  |  |  |  |  |  |  |  |
| Age group | |  | | |  | |  |  | |  | |  | |  |  |
| ≤44 | | 143 | | | 3639.77±2314.41 | | 4.90^**^ |  | | 550 | | 4000.78±3511.91 | | 19.19^***^ |  |
| 45- | | 800 | | | 4564.39±3123.06 | |  |  | | 4231 | | 4895.82±3619.89 | |  |  |
| 65- | | 567 | | | 4729.79±2680.20 | |  |  | | 3161 | | 5386.42±5316.85 | |  |  |
| 75- | | 388 | | | 4781.92±2619.12 | |  |  | | 2148 | | 5533.63±4819.89 | |  |  |
| ≥85 | | 55 | | | 4473.61±2006.64 | |  |  | | 349 | | 5717.53±6920.83 | |  |  |
| Gender | |  | | |  | |  |  | |  | |  | |  |  |
| Female | | 1172 | | | 4386.48±2439.26 | | 14.56^**^ |  | | 6120 | | 4911.27±4078.70 | | 42.24^***^ |  |
| Male | | 781 | | | 4883.83±3313.53 | |  |  | | 4319 | | 5502.62±5205.37 | |  |  |
| Admission Route | |  | | |  | |  |  | |  | |  | |  |  |
| Others | | 591 | | | 4306.80±2668.36 | | 8.23^**^ |  | | 2367 | | 5994.08±7241.17 | | 99.78^***^ |  |
| Outpatient | | 1362 | | | 4706.25±2891.72 | |  |  | | 7898 | | 4917.94±3429.17 | |  |  |
| Discharge Route | |  | | |  | |  |  | |  | |  | |  |  |
| Others | | 234 | | | 3948.70±2770.20 | | 13.53^**^ |  | | 852 | | 4874.72±5392.63 | | 3.51 |  |
| Medical discharge | | 1719 | | | 4672.04±2829.21 | |  |  | | 9570 | | 5182.30±4511.11 | |  |  |
| Disease Severity | |  | | |  | |  |  | |  | |  | |  |  |
| None | | 655 | | | 3759.61±1958.73 | | 46.42^***^ |  | | 2489 | | 4106.77±3371.32 | | 112.02^***^ |  |
| CC | | 1162 | | | 4942.69±2997.18 | |  |  | | 7003 | | 5352.73±3898.61 | |  |  |
| MCC | | 136 | | | 5509.45±3846.82 | |  |  | | 947 | | 6458.16±9216.71 | |  |  |
|  |  | |  |  | |  | | |  | |  | |  |  |  |

*: P<0.05; **: P<0.01; ***: P<0.001.

**Supplementary Table 3.** Parameters of the interruption time series analysis of inpatients with ischemic stroke (with LOS and medical expenses as dependent variables, respectively)

| Indicators | Hospital grade | Baseline level | Baseline trend | Level change | Trend change | Trend after DRG introduction |
| --- | --- | --- | --- | --- | --- | --- |
|  |  | *β0* | *β1* | *β2* | *β3* | *β1+ β3* |
| LOS (Day) | Tertiary | 7.744^***^ | -0.008 | 0.037 | -0.039^*^ | -0.047 |
|  | Secondary | 12.849^***^ | -0.366^**^ | 0.198 | -0.104^***^ | -0.470 |
| Medical expenses (RMB yuan) | Tertiary | 6466.419^***^ | -37.513 | 2.815 | 7.324 | -30.189 |
|  | Secondary | 4328.498^***^ | 18.632 | 69.467 | 26.286^***^ | 44.918 |

*: P<0.05; **: P<0.01; ***: P<0.001.

**Supplementary Table 4.** Intermittent time series analysis parameters of LOS of patients with ischemic stroke by gender, age, and disease severity

| Indicators | Hospital grade | Baseline level | Baseline trend | Level change | Trend change | Trend after DRG introduction |
| --- | --- | --- | --- | --- | --- | --- |
|  |  | *β0* | *β1* | *β2* | *β3* | *β1+ β3* |
| Gender |  |  |  |  |  |  |
| Male | Tertiary | 8.090^***^ | -0.057 | 0.114 | -0.032 | -0.089 |
|  | Secondary | 12.697^***^ | -0.349^***^ | 0.376 | -0.113^***^ | -0.462 |
| Female | Tertiary | 7.463^***^ | 0.035 | -0.003 | -0.038^**^ | -0.003 |
|  | Secondary | 12.934^***^ | -0.373^*^ | 0.08 | -0.098^***^ | -0.471 |
| Age |  |  |  |  |  |  |
| 0-44 | Tertiary | 6.199^***^ | 0.101 | 0.323 | -0.006 | 0.095 |
|  | Secondary | 9.859^***^ | -0.107 | 0.449 | -0.090^*^ | -0.197 |
| 44-64 | Tertiary | 7.481^***^ | 0.002 | 0.007 | -0.038 | -0.036 |
|  | Secondary | 13.105^***^ | -0.454^***^ | 0.505 | -0.106^***^ | -0.560 |
| 65-74 | Tertiary | 8.497^***^ | -0.110 | -0.049 | -0.049^*^ | -0.159 |
|  | Secondary | 13.505^***^ | -0.308 | 0.079 | -0.106^***^ | -0.414 |
| 75-84 | Tertiary | 7.526^***^ | 0.057 | 0.209 | -0.050^*^ | 0.007 |
|  | Secondary | 11.479^***^ | -0.177 | -0.067 | -0.100^***^ | -0.277 |
| ≥85 | Tertiary | 7.257^*^ | 0.378 | 0.071 | 0.016 | 0.394 |
|  | Secondary | 18.032^***^ | -1.399 | -0.210 | -0.081 | -1.480 |
| Disease Severity | |  |  |  |  |  |
| MCC | Tertiary | 8.806^***^ | -0.151 | -0.453^***^ | -0.046^*^ | -0.197 |
|  | Secondary | 15.735^***^ | -0.845^***^ | -0.187 | -0.083^***^ | -0.928 |
| CC | Tertiary | 8.733^***^ | -0.095 | 0.017 | -0.036^*^ | -0.131 |
|  | Secondary | 13.495^***^ | -0.361^**^ | 0.156 | -0.121^***^ | -0.482 |
| None | Tertiary | 5.783^***^ | 0.169 | 0.075 | -0.045^*^ | 0.124 |
|  | Secondary | 11.174^***^ | -0.274^*^ | 0.441 | -0.069^**^ | -0.343 |

*: P<0.05; **: P<0.01; ***: P<0.001.

**Supplementary Table 5.** Intermittent time series analysis parameters of medical expenses of patients with ischemic stroke by gender, age, and disease severity

| Indicators | Hospital grade | Baseline level | Baseline trend | Level change | Trend change | Trend after DRG introduction |
| --- | --- | --- | --- | --- | --- | --- |
|  |  | *β0* | *β1* | *β2* | *β3* | *β1+ β3* |
| Gender |  |  |  |  |  |  |
| Male | Tertiary | 7001.463^***^ | -79.131^**^ | 117.769 | 13.735 | -65.396 |
|  | Secondary | 4654.529^***^ | -10.755 | 54.363 | 36.044^***^ | 25.289 |
| Female | Tertiary | 6029.202^***^ | 4.194 | -64.495 | 11.533 | 15.727 |
|  | Secondary | 4128.745^***^ | 36.232 | 80.83 | 20.025^***^ | 56.257 |
| Age |  |  |  |  |  |  |
| ≤44 | Tertiary | 4442.705^***^ | 150.943 | 74.56283 | 64.146^*^ | 215.089 |
|  | Secondary | 3520.251^***^ | -13.987 | 255.1689 | 33.474^**^ | 19.487 |
| 45-64 | Tertiary | 6321.810^***^ | -66.704^*^ | 54.03 | 5.207 | -61.497 |
|  | Secondary | 4389.832^***^ | -11.171 | 215.618 | 22.829^***^ | 11.658 |
| 65-74 | Tertiary | 7140.566^***^ | -145.346^*^ | -22.784 | 18.935 | -126.411 |
|  | Secondary | 4547.869^***^ | 11.795 | -60.321 | 27.700^***^ | 39.495 |
| 75-84 | Tertiary | 6156.669^***^ | 107.042 | -54.476 | -11.995 | 95.047 |
|  | Secondary | 4149.152^***^ | 102.458^**^ | -41.894 | 30.602^***^ | 133.060 |
| ≥85 | Tertiary | 6635.419^***^ | 316.842 | 250.57 | 65.580 | 382.422 |
|  | Secondary | 3843.815^***^ | 87.279 | -224.405 | 46.206^*^ | 133.485 |
| Disease Severity | | |  |  |  |  |
| MCC | Tertiary | 7966.282^***^ | -112.005 | -212.774 | -19.521 | -131.526 |
|  | Secondary | 5171.367^***^ | -61.471 | -190.009 | 39.840^**^ | -21.631 |
| CC | Tertiary | 7216.951^***^ | -90.254 | -26.742 | 18.825 | -71.429 |
|  | Secondary | 4562.963^***^ | 44.056 | 41.796 | 21.883^***^ | 65.939 |
| None | Tertiary | 4752.744^***^ | 68.500 | 25.928 | -7.516 | 60.984 |
|  | Secondary | 3774.441^***^ | -12.733 | 161.617 | 31.516^***^ | 18.783 |

*: P<0.05; **: P<0.01; ***: P<0.001.
